# Dynamic assessment of exposure to air pollution using mobile phone data

**DOI:** 10.1186/s12942-016-0042-z

**Published:** 2016-04-21

**Authors:** Bart Dewulf, Tijs Neutens, Wouter Lefebvre, Gerdy Seynaeve, Charlotte Vanpoucke, Carolien Beckx, Nico Van de Weghe

**Affiliations:** Department of Geography, Ghent University, Krijgslaan 281, S8, 9000 Ghent, Belgium; Research Foundation Flanders, Egmontstraat 5, 1000 Brussels, Belgium; VITO, Boeretang 200, 2400 Mol, Belgium; Proximus, Koning Albert II-laan 27, 1030 Brussels, Belgium; IRCEL, Kunstlaan 10-11, 1210 Brussels, Belgium

**Keywords:** Air pollution, Dynamic assessment, Exposure, Mobile phone data, Travel patterns

## Abstract

**Background:**

Exposure to air pollution can have major health impacts, such as respiratory and cardiovascular diseases. Traditionally, only the air pollution concentration at the home location is taken into account in health impact assessments and epidemiological studies. Neglecting individual travel patterns can lead to a bias in air pollution exposure assessments.

**Methods:**

In this work, we present a novel approach to calculate the daily exposure to air pollution using mobile phone data of approximately 5 million mobile phone users living in Belgium. At present, this data is collected and stored by telecom operators mainly for management of the mobile network. Yet it represents a major source of information in the study of human mobility. We calculate the exposure to NO_2_ using two approaches: assuming people stay at home the entire day (traditional static approach), and incorporating individual travel patterns using their location inferred from their use of the mobile phone network (dynamic approach).

**Results:**

The mean exposure to NO_2_ increases with 1.27 μg/m^3^ (4.3 %) during the week and with 0.12 μg/m^3^ (0.4 %) during the weekend when incorporating individual travel patterns. During the week, mostly people living in municipalities surrounding larger cities experience the highest increase in NO_2_ exposure when incorporating their travel patterns, probably because most of them work in these larger cities with higher NO_2_ concentrations.

**Conclusions:**

It is relevant for health impact assessments and epidemiological studies to incorporate individual travel patterns in estimating air pollution exposure. Mobile phone data is a promising data source to determine individual travel patterns, because of the advantages (e.g. low costs, large sample size, passive data collection) compared to travel surveys, GPS, and smartphone data (i.e. data captured by applications on smartphones).

## Background

A large body of evidence indicates that exposure to air pollution causes various acute and chronic health effects, such as respiratory and cardiovascular diseases [1–9]. Approximately 2 million deaths worldwide are caused by air pollution annually [10]. Mainly black carbon (BC), particulate matter (PM), and nitrogen dioxide (NO_2_) are identified as culprits of negative health effects.

Current health impact assessments and epidemiological studies examining exposure to air pollution often only take the air pollution concentration at the home location into account [11–17]. Such static approach does not incorporate individual travel patterns and may lead to a bias in exposure and health assessments [18–23].

Detailed information on travel patterns is thus needed to obtain more dynamic estimates of the exposure to air pollution. Previous research showed an increase in exposure to air pollution by incorporating individual travel patterns [24], but the outcome depends on the air pollution concentration at the home location [25]. To assess individual travel patterns, often self-reported household travel surveys are used [26]. Major disadvantages of this approach are the large non-response rate [27], non-representative samples [28], and high costs [26]. Alternatively, mathematical models of travel patterns can be used [18, 24, 29]. This approach allows to draw more quantitative conclusions from a larger population size, but results are however only valid for situations similar to those for which their initial parameters were estimated. More recently, Global Positioning Systems (GPS) [25, 30] or smartphone data [31, 32] were used to provide detailed information on people’s travel patterns. However, data collection with GPS or smartphone devices is often intensive for both researchers and participants, expensive and only a limited number of people can be tracked.

To overcome the limitations of travel surveys, travel models and GPS/smartphone data, mobile phone data can be used to derive information on individual travel patterns. At present, this kind of data is collected and stored by telecom operators mainly for management of the mobile network. However, it represents a major source of information in the study of human mobility. This data is continuously available, does not need additional costs to collect, and is often available for millions of phone users. With over 6 billion mobile subscriptions globally and a growing awareness of telecom operators of the potential, this data source offers a wide range of applications and research possibilities [33]. However, the number of studies published with such data is limited up to now because of privacy issues and problems accessing the data [34]. Previous studies using this type of data mostly analyse population densities [35–37], tourism [38, 39], and mobility [33–35, 40–44]. To our knowledge, no studies have used mobile phone data to dynamically estimate the exposure to air pollution.

This research will add knowledge to the existing strand of literature by calculating the exposure to air pollution using mobile phone data of more than 5 million people in Belgium. Our main objective is to bring evidence on how this innovative, underused data source can offer more dynamic estimations of the exposure to air pollution. Further, we explore how using daily averaged and hourly air pollution concentrations influences the results.

## Methods

### Data

#### Mobile phone data

Mobile phone data (or passive mobile positioning data) is based on signalling information that is exchanged between mobile devices and the mobile network. When using the mobile network, there is a flow of signalling information between the device and the mobile network. The mobile device switches to the antenna with the strongest radio coverage, which is typically the closest one. The signalling messages contain an indication of the antenna in use.

**Fig. 1:**
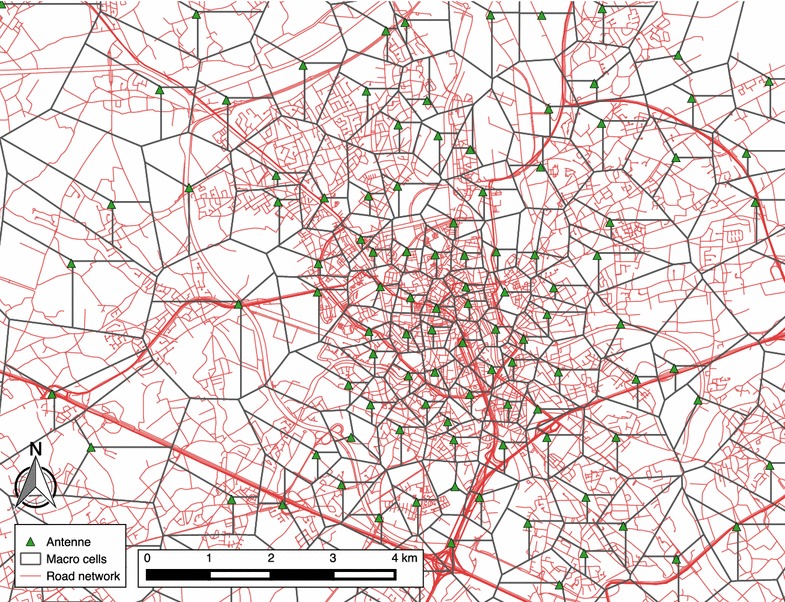
Map showing the macro cells of the region of Ghent, overlaid on the road network

**Fig. 2:**
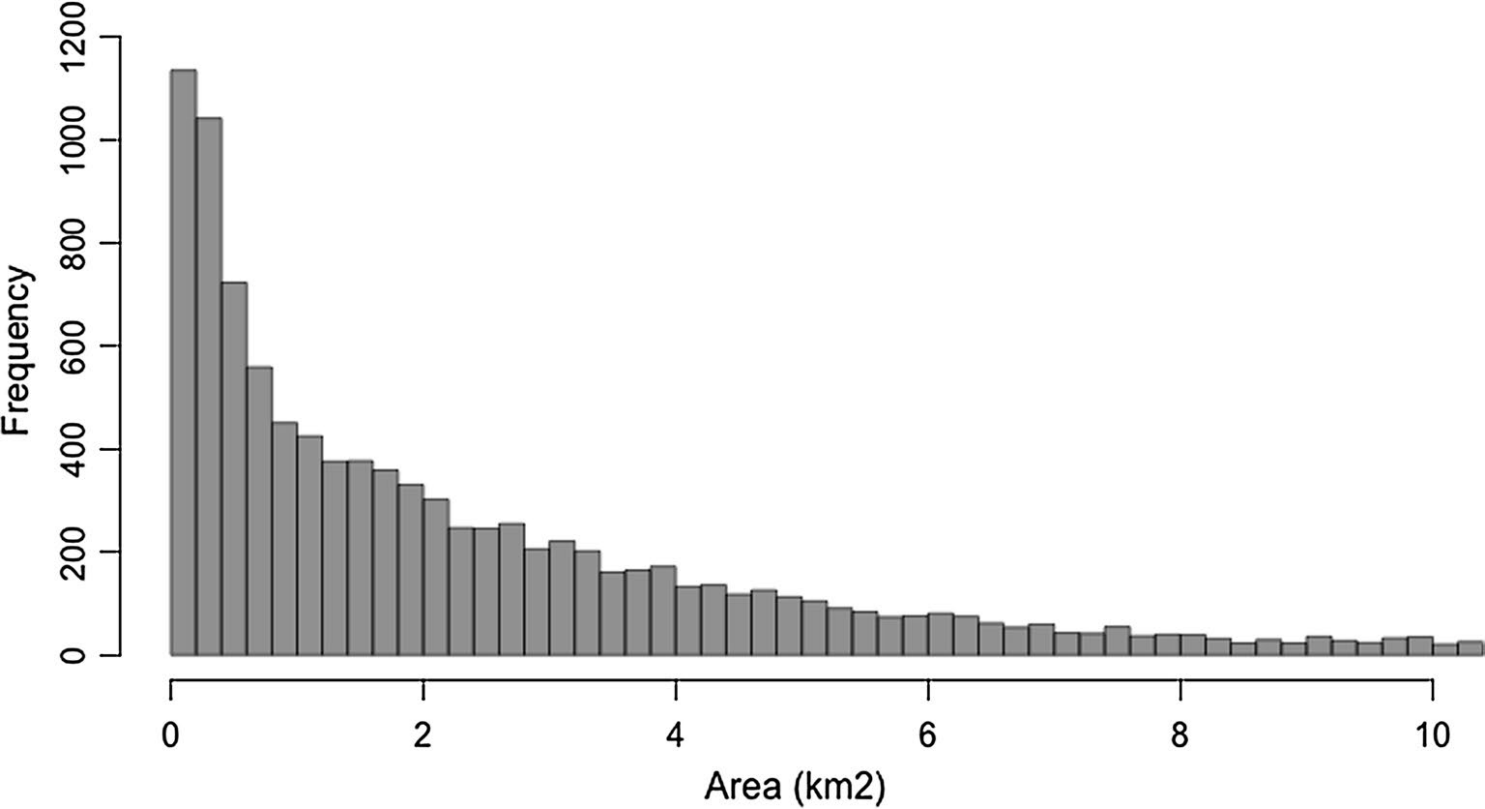
Histogram showing the area (km^2^) of the macro cells

The data used for this study is available from probes installed in the Proximus network, which capture this information. We have data available from more than 4000 antennae sites. On each antenna site there are typically three or four antennae, delivering network coverage in diverged directions. As sites can be equipped with 2G, 3G and 4G technologies in different frequency bands, we make abstraction of the different technologies and group all cells that are co-located on the same antenna site and cover the same sector to considerably reduce complexity. This leads to more than 10,000 macro cells covering the entire country of Belgium. The mobile phone location is thus available at the precision of these macro cells, with each cell having its own, unique geographical coverage area and identity code. Figure 1 shows the antennae with the associated macro cells for the region of Ghent, overlaid on the road network. Because of the higher capacity needs, macro cells are smaller in urban areas and larger in rural areas. Figure 2 shows a histogram of the area of the macro cells. Some macro cells are larger than 10 km^2^ (with a maximum of 49 km^2^), but 50 % of them have an area smaller than 2 km^2^.

Data is collected from more than 5 million users of the Proximus network, which are representative for the Belgian population [45]. In Belgium, Proximus has a market share of about 41 %, which is higher than the other Belgian telecom operators: Mobistar (27 %), Telenet (14 %), Base (11 %), and others (7 %) [46].

The network probing system collects from all active users. Each data point consists of an anonymised user ID, the date and time of the transaction, the cell where the transaction occurred, and the transaction type. The following transaction types are possible: (a) turning on and off the phone; (b) setting up, maintaining and terminating calls; (c) sending and receiving text messages; (d) setting up, maintaining and terminating data sessions; (e) location update (when changing from location area; a location area is a group of cells of which there are approximately 65 in the Proximus network); (f) periodic location update (automatic update every 3 h when there is no activity). For this study, we used mobile phone data for both 1 week and 1 weekend day: Thursday October 8 and Saturday October 11, 2015. Because of regulation terms we had limited access to the data, but these 2 days were chosen to be as representative as possible, in terms of weather conditions for the time of the year, and travel behaviour (e.g. no holidays). No data of the home location was available due to privacy issues. Therefore, we used the location of the users at 4 am as a proxy for their home location (hereafter called reference location), since it is assumed most of the people are at home at that time.

Privacy issues of using mobile phone network data are a major concern of phone owners, telecom operators, researchers, and the general public. Because of this, no personal information is linked to the mobile phone data, and IDs that can link directly to individuals are removed. Individual exposure measures were aggregated to postal code level for mapping purposes.

#### Air pollution data

We focus on NO_2_, an understudied pollutant that can cause an increase in pulmonary morbidity, a worsening of obstructive lung disease, and a higher susceptibility to airway infections [9, 47, 48]. Hourly NO_2_ concentration data (in μg/m^3^) for Belgium was provided by the coupled RIO-IFDM model [49]. This model couples the land use regression model RIO, the road emissions model MIMOSA4 (taking into account COPERT4 emission functions, vehicle fleet and vehicle counts), and the Gaussian plume model IFDM. The latter is used to incorporate large concentration variations close to the major air pollution sources, such as roads and point sources. The model has been validated extensively for the discussed region [49, 50]. Hourly air quality measurements are provided by the Belgian Interregional Environment Agency [51].

**Fig. 3:**
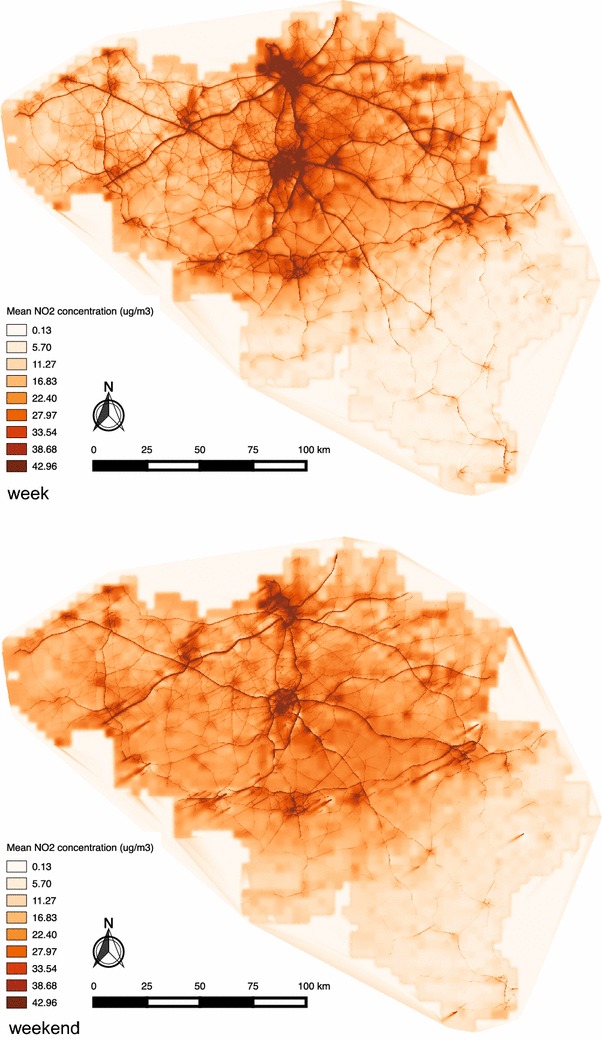
Map showing the mean NO_2_ concentration for the entire country of Belgium, for both Thursday October 8 and Saturday October 11 2015

In line with the mobile phone data, NO_2_ concentration patterns in Belgium were modelled during 2 days. NO_2_ concentration levels varied from 3 to 63 μg/m^3^ on the weekday (Thursday October 8, 2015) and from 5 to 54 μg/m^3^ on the weekend day (Saturday October 11, 2015). Figure 3 shows the mean NO_2_ concentration for the entire country of Belgium, for both Thursday October 8 and Saturday October 11, 2015.

### Data processing

**Fig. 4:**
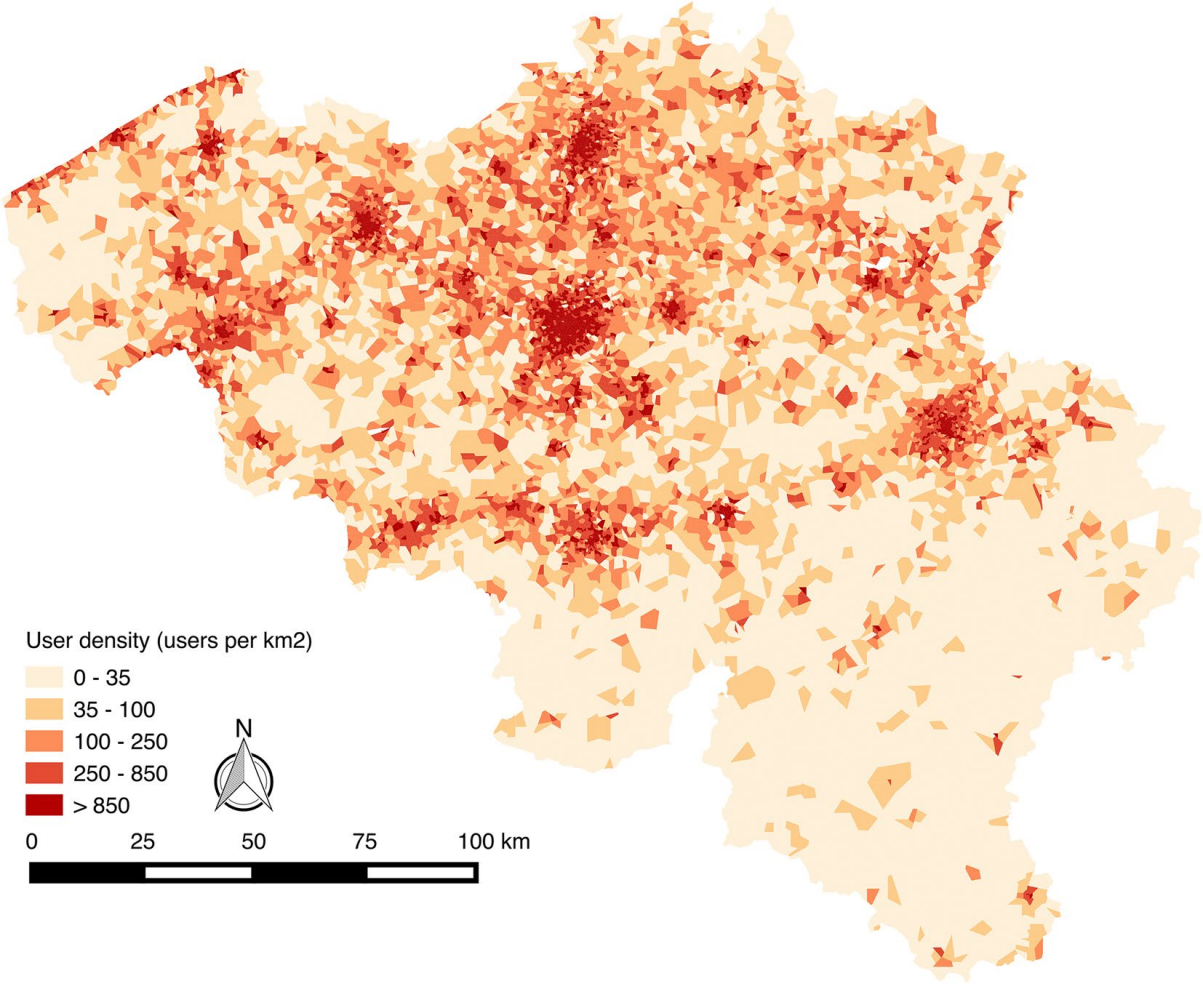
User density per cell on October 8 2015 at 12 am UTC

Mobile phone data are collected and stored by the telecom operator, mainly for management of the mobile network and technical operations. Because each user in the mobile network has a different mobile activity, the temporal resolution of the data varies. The last known position (cell) of each user was used at a temporal resolution of 15 min. We assume that, when there is no new data point within 15 min, the user is still at the same location as before. As an example, Fig. 4 shows the user density (number of users per cell divided by the cell area) of Thursday October 8, 2015 at 12 am UTC.

Proximus has 5,574,000 active costumers [52]. Active costumers are costumers who have made or received at least one call, or sent or received at least one message in the last 3 months, or if at least one data connection has been made on the last month. From the initial dataset, users are omitted if:

- they are international users,
- their data relate to machine to machine transactions (e.g. car kits; to avoid duplicate data),
- their travel patterns exceed the borders of Belgium during the selected days (no air pollution concentration available),
- they have no known position from 1:00 until 4:00 in the morning (necessary to derive the reference location).

This results in a dataset of 3,465,917 users on the weekday and 3,495,453 on the weekend day.

The point dataset of the NO_2_ concentration is triangulated to a 50 × 50 m grid, using the SAGA ‘gridding triangulation tool’ in QGIS. Following, we calculate the average NO_2_ concentration per cell using the SAGA ‘grid statistics for polygons tool’ in QGIS to combine this with the location data. The mean NO_2_ concentration per cell is 29.36 μg/m^3^ on the weekday and 27.32 μg/m^3^ on the weekend day, with a mean standard deviation per cell of respectively 3.62 μg/m^3^ and 2.73 μg/m^3^.

The location of the users is combined with the air pollution concentration, to calculate the exposure to air pollution. The air pollution data is in Coordinated Universal Time (UTC), and the mobile phone data is in local time (UTC+1). Therefore, we have an overlap of 23 h (92 quarters) per day, and are thus able to combine the datasets from 0 am UTC to 11 pm UTC. The exposure to NO_2_ is calculated using either a static or dynamic location. For the static approach, we use the cell where the user is at 4 am UTC as their reference location. For the dynamic approach, we use the exact cell where the user is, at a temporal resolution of 15 min. Additionally, we use the NO_2_ concentration per cell in two different ways. We either use the hourly concentration or the daily averaged concentration per cell. This results in four possible average air pollution concentrations each user is exposed to during the day.

### Data analyses

First, having these four average air pollution exposure values for 2 days, we check what the influence of using hourly air pollution concentrations (hour) is on the exposure to air pollution, compared to using the daily average air pollution concentration (day). Second, we compare the effect of using the reference location of the user (static) with taking into account the actual location of the user (dynamic) on the calculated exposure to air pollution. Third, this comparison is also analysed geographically.

The data was statistically analysed using R 3.2.2™. To check for significant differences between the approaches, paired-samples *t* tests (hour vs. daily averaged air pollution concentration, static vs. dynamic approach, week vs. weekend days) were performed. The data does not have to be tested for normality, because of the large sample size [53]. Statistical significance was set at *p* < 0.05. Geographical analyses were performed in QGIS 2.12™. Averages of individual exposure values were calculated per municipality and visualised using choropleth maps.

## Results

**Fig. 5:**
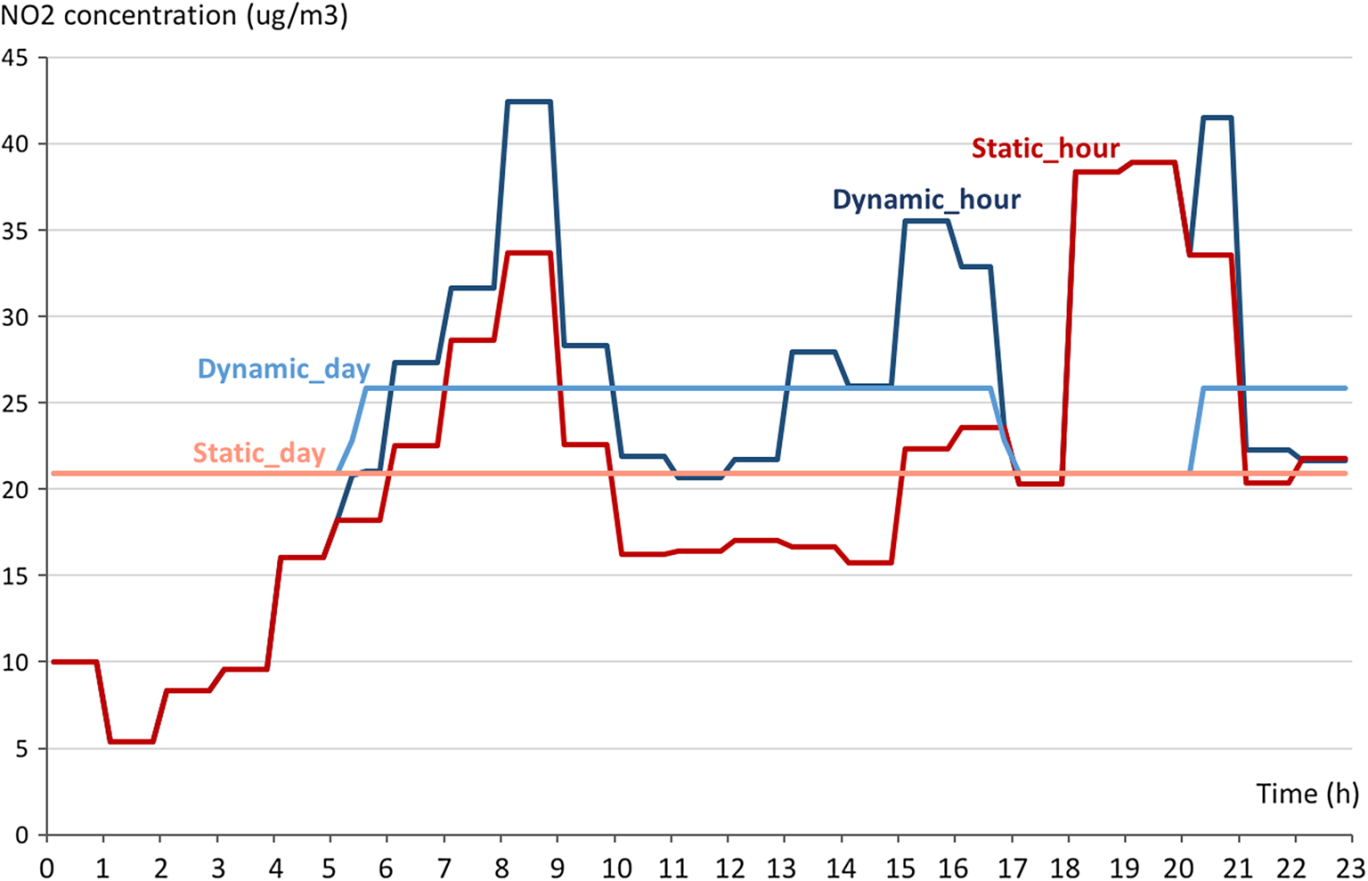
Exposure to NO_2_ during the weekday for a random user, using the four different approaches (static_hour, dynamic_hour, static_day, dynamic_day)

To gain insight into the origin of the four average values per user, Fig. 5 shows the exposure to NO_2_ during the weekday for a random user, calculated statically and dynamically, with both hourly and daily averaged NO_2_ concentrations. Using hourly NO_2_ concentrations leads to a higher level of detail of the exposure to air pollution. However, since we calculate the average exposure to air pollution per day, this has limited effects on the results. It is also clear that taking into account actual travel patterns (instead of assuming the person stays at the reference location) leads to a different exposure to air pollution. In this case, the person spends time in cells with a higher NO_2_ concentration than at his or her reference location.

The mean NO_2_ exposure per person was calculated statically and dynamically, using hourly NO_2_ concentration values for both the week and weekend day, and is presented in Table 1. The significance of the differences was tested using multiple paired-samples *t* tests (daily averaged vs. hourly NO_2_ concentrations, static vs. dynamic, week vs. weekend).

**Table 1:** Mean exposure to NO_2_ per person, calculated statically and dynamically, using hourly and daily averaged NO_2_ concentrations, for both the weekday (*n* = 3,465,917) and weekend day (*n* = 3,495,453)

Method	Mean NO_2_ exposure (μg/m^3^) [σ_x_]			
	Weekday		Weekend day	
	NO_2_ per hour	NO_2_ per day	NO_2_ per hour	NO_2_ per day
Static	29.69 [12.03]	29.69 [12.03]	27.47 [8.58]	27.47 [8.58]
Dynamic	30.96 [11.26]	30.83 [11.25]	27.59 [7.99]	27.57 [8.01]
Difference	1.27 [5.02]	1.14 [4.43]	0.12 [2.82]	0.10 [2.41]

### Comparison of using hourly or daily averaged air pollution concentrations

From Table 1, we observe practically no difference in the mean NO_2_ exposure calculated with hourly and daily averaged NO_2_ concentrations for the static approach, which is expected. For the dynamic approach, we observe a small significant difference (*p* < 0.001) only during the week. Here, the calculated NO_2_ exposure is 0.13 μg/m^3^ (0.4 %) higher when using hourly values compared to when using daily averaged values.

### Comparison of the static and dynamic calculation of the exposure to air pollution for a week and weekend day

To compare the static with the dynamic approach, we will only consider the values calculated with the hourly NO_2_ concentrations, since this way the highest level of detail is obtained.

**Fig. 6:**
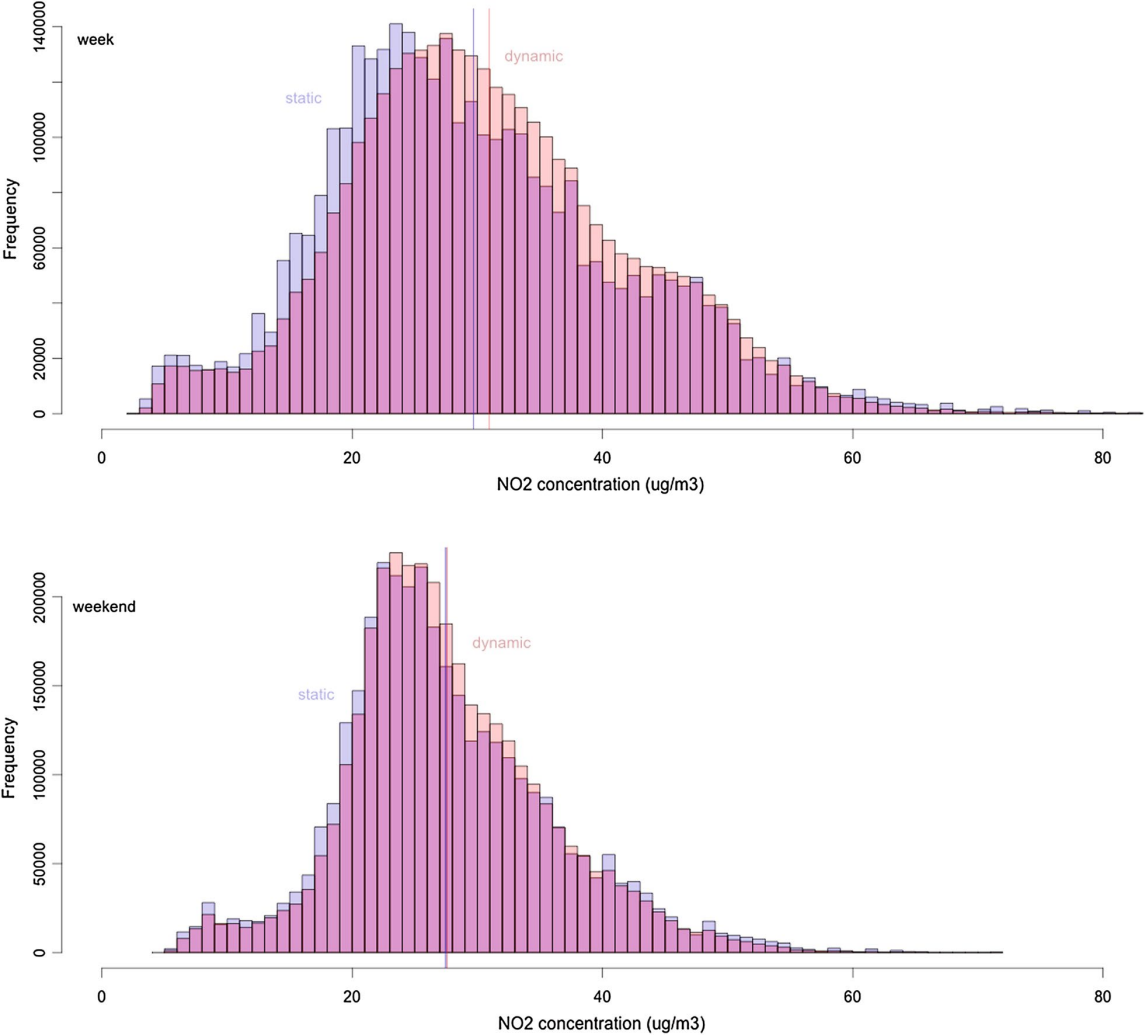
Histogram of the average NO_2_ concentration that the users are exposed to using the static and dynamic approach, for the week (*n* = 3,465,917) and weekend day (*n* = 3,495,453), including the mean reference line for both approaches

Table 1 shows that by incorporating individual travel patterns (dynamic), the mean exposure to NO_2_ increases with 1.27 μg/m^3^ (4.3 %) on the weekday and with 0.12 μg/m^3^ (0.4 %) on the weekend day, compared to assuming the person stays at the reference location (static). These values are all significantly different from each other (*p* < 0.001). Figure 6 combines the static and dynamic approach for the two days in a histogram. It is clear that during the week, there are more users who experience an increase in exposure to NO_2_. During the weekend, the values are more central and the increase in NO_2_ exposure is less pronounced. During the week, 12.4 % of the users have no change, 54.5 % have an increase, and 33.1 % have a decrease. During the weekend, 20.1 % have no change, 43.3 % have an increase, and 36.6 % have a decrease.

**Fig. 7:**
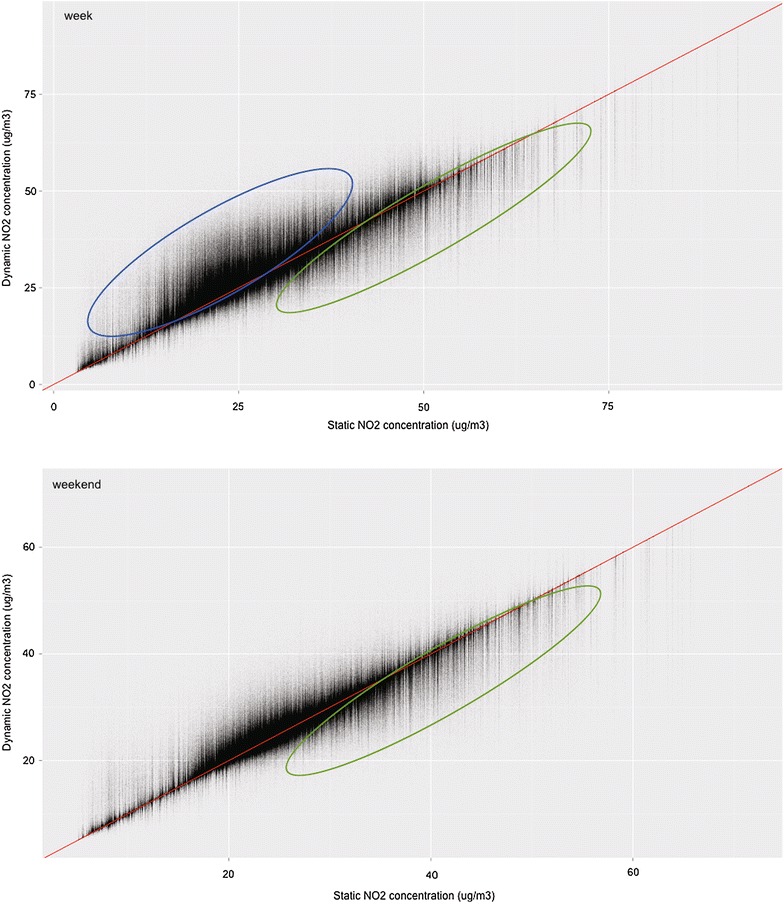
Scatterplot of the exposure to NO_2_ calculated with the static and the dynamic approach, for the week (*n* = 3,465,917) and weekend day (*n* = 3,495,453)

Figure 7 shows a scatterplot of the exposure to NO_2_ calculated statically and dynamically, for both the week and weekend day. During the week, it is clear that users with a low NO_2_ exposure calculated statically (thus with a low average NO_2_ concentration at the reference location) experience a strong increase in NO_2_ exposure when dynamically calculated (indicated in blue), a pattern that is less pronounced during the weekend. This is also true the other way round: users with high NO_2_ concentrations at the reference location experience a decrease in NO_2_ exposure when their travel patterns are considered (indicated in green), which is also observed during the weekend.

### Geographically analysing the comparison between the static and dynamic calculation of the exposure to air pollution

Next to these statistical analyses, we also performed geographical analyses on the comparison between the static and dynamic approach to calculate the NO_2_ exposure. Here, again only hourly NO_2_ concentrations were used in the calculations.

**Fig. 8:**
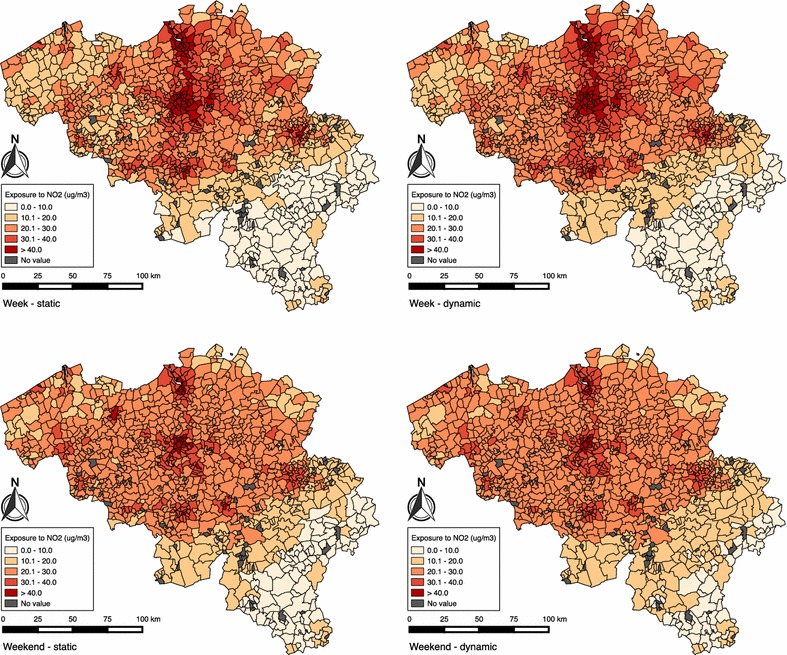
Maps of Belgium, showing the statically and dynamically calculated exposure to NO_2_, for the week (*n* = 3,465,917) and weekend day (*n* = 3,495,453)

**Fig. 9:**
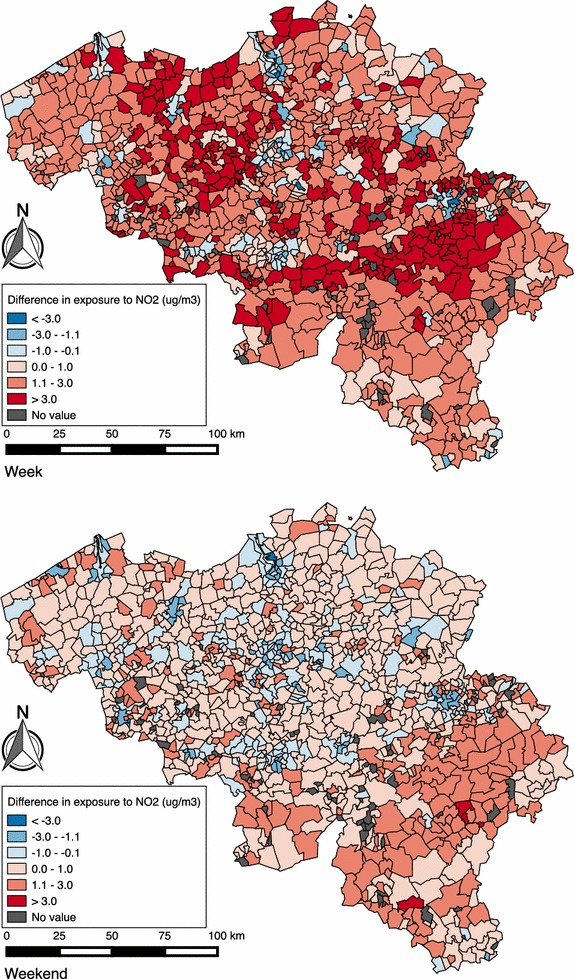
Maps of Belgium showing the difference between the statically and dynamically (dynamic minus static) calculated exposure to NO_2_, for both the week (*n* = 3,465,917) and weekend day (*n* = 3,495,453)

Figure 8 shows the average exposure to NO_2_ per municipality, for both the week and weekend day, calculated statically and dynamically with hourly NO_2_ concentrations. Figure 9 shows the difference between the average exposure to NO_2_ calculated with static and dynamic approach (dynamic minus static), using hourly NO_2_ concentrations, per municipality for both the week and weekend day. During the week, there is a large increase mostly in the municipalities surrounding larger cities (Brussels, Antwerp, Ghent) and a decrease in these larger cities. During the weekend we observe a similar pattern, but with lower increases and more decreases in the difference between the static and dynamic approach.

## Discussion

### General discussion

First, our study shows that using daily averaged instead of hourly NO_2_ concentrations leads to only a 0.4 % decrease in dynamically calculated exposure to NO_2_ in our analyses. If hourly data is available, it is preferred to use it [18, 31]. If, however, no detailed hourly NO_2_ concentration data is available, the impact will be limited.

Second, our study supports the findings of several recent studies stating the importance of incorporating individual travel patterns in estimating air pollution exposure [18, 20–23, 25], an issue sometimes overlooked [11]. Mobile phone data makes it possible to estimate individual travel patterns to use in health impact assessments and epidemiological studies. We observe a mean increase in NO_2_ exposure of 4.3 % during the weekday and 0.4 % during the weekend day when incorporating individual travel patterns, which means that current health impact assessments underestimate the exposure to NO_2_ and the related acute and chronic health effects. These increases were also found in previous research, where integrating time-activity information lead to a 1.2 % increase air pollution exposure than when assuming people are always at their home location [24]. We observed an increase or decrease in NO_2_ exposure for respectively 54.5 and 33.1 % of the users because of their travel patterns during the week. In the weekend, respectively 43.3 and 36.6 % of the users experience an increase or decrease in exposure. Thus, people tend to make more trips to areas that are less polluted than their reference location in the weekend than during the week. During the week, people living in areas with a low NO_2_ concentration undergo an increase in NO_2_ exposure because of their travel patterns (going to work in a more polluted area) whereas people living in highly polluted areas undergo a decrease in NO_2_ exposure, which is similar to our previous study [25].

Third, concerning the geographical analysis, our study reports that people living near Brussels are most exposed to NO_2_ both during the week and the weekend, because of the highest density of air pollution sources (industry and roads), and Brussels being one of the most congested cities in Europe [54, 55]. People living in the south of Belgium are least exposed to NO_2_. Mainly people from municipalities around larger cities experience an increase in exposure to air pollution during the week because of their travel patterns (going to work in these cities). The average difference for people living in these cities is negative since people working in the city do not experience any change and people working outside the city experience a decrease in NO_2_ exposure. During the weekend, we observe lower increases and more decreases in NO_2_ exposure because of individual travel patterns, because during the weekend people tend to visit more areas with lower air pollution concentrations than at the reference location. In more rural areas (e.g. the south of Belgium) there is an increase in NO_2_ exposure during the weekend when incorporating travel patterns, because every trip people living in this area make leads to an increased NO_2_ exposure due to the low air pollution concentration at people’s reference location.

### Strengths and limitations

This study has several strengths compared to similar studies. First, to our knowledge, this is the first study that combines mobile phone data with air pollution concentration data to dynamically estimate the exposure to air pollution. Using mobile phone data has several advantages above GPS data or questionnaires: no additional costs have to be made to collect the data, a very large number of people can be traced because of the wide adoption of the mobile phone, the data collection is passive so people are not disturbed and does not influence the battery of their mobile devices, and they are tracked without them knowing so they don’t change their normal behaviour. Numerous practical applications can be developed based on the presented method, both for individual as on a community level. Policy makers can for example be interested to follow-up the average population exposure indicator or to assess the impact of a policy measure such as on the exposure. However, it is not easy to access mobile phone data. Concerning privacy issues, good agreement with the telecom operator is needed, as well as a clear understanding of the data use [33, 38]. A second strength of the current study involves the type of location data that were used. Previous studies using mobile phone data only collected a location when users made a phone call or sent a text message. In our study, we additionally locate users when turning the phone on or off, during a data session, when changing location area, or when periodically updating the location area by the telecom operator. This approach significantly improves the spatial accuracy of all the users but also includes relevant information on non-frequent callers in the population. Third, using modelled air pollution concentrations instead of personal measurements offers nation-wide data on a detailed geographical scale [18, 24] and is easier to generate than personal measurements [19]. Fourth, we used both the hourly and daily averaged NO_2_ concentrations in contrast to our previous research [25], making a comparative analysis possible. Using daily averaged values does not alter the results extremely, since we calculated daily exposure values. It is however preferred to use the hourly values when available, to obtain more accurate results.

Apart from these strengths, this study also has some limitations that open up interesting avenues for future work. First, we only used air pollution concentration and individual travel data for two days. Despite the fact that these days were chosen to be as representative as possible, it would be better to use more data, e.g. for an entire week, month or even year. Also, in order to assess the associated health impacts, more data is required. Second, following the privacy issues of mobile phone data, it is difficult to combine this data with personal sociodemographic variables or other semantic information (e.g. transport purpose and trip mode), which limits the analysis possibilities. Future research could try to deduce the sociodemographic characteristics from the most likely living place (possible to determine using long-term location data) to get an idea of the socioeconomic status of the users. These privacy issues could be addressed by using privacy-enhancing technologies [56]. One possible solution is to slightly obfuscate the location of the user, while keeping enough information to perform satisfying analyses [57]. An other possibility is that telecom providers could ask for an opt-in consent from their costumers to make use of their location data for scientific research and try to build a trust relationship with them, and build services where costumers benefit from. Third, since we had no information on the user’s home location, we used the location at 4 am as a proxy for their home location (and use it as reference location). A better solution would be to use mobile phone data from a longer period (e.g. 1 month), to make a more accurate estimate of the most likely living location. Fourth, the spatial resolution of mobile phone data is limited to that of the used cells, which is low compared to the spatial accuracy of GPS data. Because of this low spatial resolution, local differences in air pollution concentration (e.g. near roads) may not be taken into account. This might mean that the exposure to air pollution is probably even higher because of people spending time in traffic [21]. On the other hand, the pollution concentration gradients in the rural areas, where the macro cell size is larger, are in general very small. As a result, smaller rural macro cells, if available, would not increase the accuracy of our results. The spatial resolution of the data could be increased by applying triangulation [36, 40].

## Conclusions

Hourly air pollution concentrations are preferably used over daily averages to maximise the level of detail when combining air pollution with individual travel patterns. This study shows that for epidemiological studies and exposure assessments, it is relevant to incorporate individual travel patterns to estimate the exposure to air pollution. The change in exposure to air pollution depends on the air pollution concentration at the reference location and someone’s individual travel patterns, but on average we found an increase of 4.3 % in the exposure to NO_2_ during the week and 0.4 % during the weekend. People living in and near large cities are most exposed to NO_2_. However, people from other areas experience a higher increase in NO_2_ exposure when taking their travel patterns into account. Mainly people living in municipalities surrounding larger cities have an increase in NO_2_ exposure because they work in these cities. Aside from privacy issues, we strongly believe that using mobile phone data has several advantages (e.g. low costs, large sample, passive data collection) over travel surveys, GPS, and smartphone data. Especially for air pollution research the applications of using mobile phone data are numerous. Policy makers can use this information to assess the impact of air pollution on the population. Also, they can analyse the impact of a certain policy measure or occurring events (e.g. festivals, strikes) on the individual travel patterns and assess the associated impacts on exposure to air pollution. Mobile phone data is therefore a promising data source for air pollution research.
